# Role of extracellular vesicle–mediated neurodegeneration in substance use disorders

**DOI:** 10.1016/j.cophys.2025.100826

**Published:** 2025-04-10

**Authors:** Mohit Kumar, Arnab Saha, Agasou Alfonso Rameau, Susmita Sil, Shilpa Buch

**Affiliations:** Department of Pharmacology and Experimental Neuroscience, University of Nebraska Medical Center, Omaha, NE 68198-5880, USA

## Abstract

Substance use disorders (SUDs) remain a complicated and widespread public health problem, characterized by obsessive drug and alcohol use, despite adverse consequences. Emerging research suggests that the extracellular vesicles (EVs) play a critical role in mediating drug addiction and several neurodegenerative processes associated with SUDs. EVs, which include exosomes, microvesicles, and apoptotic bodies, are lipid-bilayered vesicles that facilitate intracellular communication throughout the host by shuttling bioactive molecules, such as proteins, lipids, DNA fragments, and RNA, including both coding and noncoding RNAs across recipient cells. The current review is a comprehensive analysis highlighting the potential role of EVs in the onset and progression of SUD, specifically in the context of cocaine, cannabis, methamphetamine, opiates, alcohol, and tobacco. The goal is to offer valuable insights into the underlying mechanism(s) involving EVs in the pathogenesis of SUD, ultimately paving the way for new therapeutic avenues.

## Introduction

Extracellular vesicles (EVs) are lipid-bilayered heterogeneous structures released from a variety of cell types that facilitate intercellular communication by carrying diverse macromolecules (cargos), such as proteins, lipids, DNA, and RNA (comprising mRNAs and noncoding RNAs, including micro (mi)RNA and short interfering (si)RNAs) [1]. The contents of EVs reflect the intracellular environment of the donor host cell(s). EVs, known to shuttle diverse cargoes and act as mediators of intercellular communication, are gaining increased attention in various biological fields. Based on their biogenesis, EVs are categorized into three broad types: 1. Exosomes (30–150 nm), 2. Microvesicles (100–500 nm), and 3. Apoptotic bodies (500–5000 nm) [2]. Herein, we collectively refer to all types of subvesicles as ‘EVs’. EVs play an important role in cell-to-cell communication by shuttling various molecules [3–8], signal transduction mediators [9], and antigen presentation moieties [10], across neighboring and distant cells. EVs derived from mesenchymal stem cells have been used extensively for their therapeutic properties, in the area of inflammation, anticancer therapy [11], and tissue repair. Interestingly, the ability of EVs to carry diverse cargoes across cells has also been used to exploit their role as conduits for potential drug delivery systems [12,13]. The ability of EVs to cross the blood–brain barrier (BBB) has made them appealing for delivering drugs to the brain in the field of neurobiology [14]. EVs can be engineered to pass through the BBB for drug delivery [15]. Recent research has highlighted the role of several EV-based noncoding regulatory miRNAs in the host response to various addictive substances, such as cocaine [16,17], cannabinoids [18], nicotine [19,20], alcohol [21,22], and opioids [23]. It is thus plausible that EVs and their contents can significantly influence the onset and progression of addiction to misused drugs. Extensive, in-depth research is warranted to fully understand how EVs impact addictive pathways and how addictive substances, in turn, affect EV release, uptake, and cargo composition. This mini-review aims to summarize the existing literature that explores the involvement of EVs in the neuropathogenesis of addiction and SUDs.

## Cocaine

Cocaine is a psychostimulant that enhances brain activity by inhibiting the reuptake of dopamine at synapses, primarily through its interaction with the dopamine transporters [24]. Cocaine exposure has been shown to increase the release of EVs from neuroglia-blastoma cells via disruption of sigma-1 receptor (Sig-1R)–ARF6 (ADP-ribosylation factor 6) complex in a concentration and time-dependent manner [25]. Additionally, in EVs derived from human microglial cell line HMC3 (HMC3-EVs), cocaine was shown to decrease the expression of CD63 (Exosome marker, critical for cellular signaling) and dectin-1 (Involved in chemokine and cytokine production) while increasing the expression of histone H2A.x (an apoptotic marker), which, in turn, interferes with cell-to-cell communication and cellular signaling [26]. A recent study demonstrated that cocaine altered the protein composition and ATP generation capacity in mitovesicles (mitochondrial origin non-exosomal EVs) [27]. Furthermore, cocaine was also shown to boost EV release from HIV-infected macrophages, dendritic, and T cells. EVs from HIV-infected macrophages demonstrated increased presence of viral genes and were also shown to induce the release of proinflammatory cytokines when exposed to human brain microvascular endothelial cells, thus implicating the role of infected macrophage EVs in mediating barrier breach and augmenting neuroinflammation [28]. Numerous investigations have also demonstrated the therapeutic potential of EVs [29,30]. For example, a study from our lab reported that intranasal delivery of mir-124 (regulator of microglial quiescence) loaded EVs in cocaine-administered mice resulted in microglial uptake of the EVs leading to dampening of cocaine-mediated microglial activation and, in turn, reduced neuroinflammation [31].

## Cannabis

Plant *Cannabis sativa* (marijuana) contains tetrahydrocannabinol (THC) and cannabidiol (CBD), known as cannabinoids. Cannabinoids exert various effects, including analgesic, anti-inflammatory, and anxiolytic effects, involving the cannabinoid receptors type 1 & 2 (CBR1 & 2), which are found in neuronal and glial cells [32]. Repeated cannabinoid exposure has been shown to lead to glial activation and the development of cannabinoid use disorder [33]. In another study, it was demonstrated that simian immunodeficiency virus (SIV) infection alone, or in SIV-infected animals chronically exposed to delta-9-THC, resulted in the release of bioactive blood extracellular vesicles (BEVs) that could trigger unique cellular structural changes and signaling pathways. Additionally, BEVs derived from SIV-infected macaques mediated pathogenic processes encompassing signal transduction pathway alterations, enhanced cell migration and adhesion to the extracellular matrix substrate collagen, and modifications in cytoskeletal dynamics. In contrast, THC (THC/SIV) stimulated BEVs suppressed SIV infection, thus underscoring the anti-inflammatory properties of cannabinoids [34]. It is likely that these BEVs could pass across the BBB to exert their effects [35]. Another mechanism by which HIV infection can impact the EV cargo release could be attributed to the ability of HIV-1 proteins and RNA to mediate dysregulated autophagy. Autophagic disturbances could impede viral protein breakdown and, as a result, lead to viral protein product build-up in the cytoplasm, ultimately contributing to the EVs cargo content and progression of HIV-associated neurocognitive disorders. As an example, in monocytes, CBD, which mediates dysregulated autophagy, was shown to dose-dependently reduce both HIV-1 transcription and EV-associated viral RNA and protein release [36].

## Methamphetamine

Methamphetamine (METH) is a psychostimulant that can cross the BBB and damage neurons and glial cells [37–39]. METH exposure has been shown to activate oxidative and inflammatory pathways and promote the release of endothelial-derived microparticles (EMP), a subtype of EV [40]. Human METH users have been shown to exhibit lower levels of EV miRNA expression and higher EV numbers in the plasma. In addition, METH is also shown to regulate the expression of eight miRNAs present in plasma EVs, of which, three miRs — miR628, miR3825p, and miR301a3p — have been found to be closely linked to neuroinflammation and synaptic plasticity [41]. In self-administered rats and rhesus macaque models of METH dependence, as well as in the *in vitro* immortalized microglial cells, METH was shown to enhance the biogenesis of both brain-derived and microglial-derived EVs, as well as the secretion of EV-miR29a [42]. Furthermore, the uptake of EV-miR29a by microglia and neurons was found to result in its binding and activation of the toll-like receptors (TLR)-7, in turn, leading to the induction of proinflammatory cytokines, while also inducing synaptodendritic injury [42]. Similarly, METH was also shown to enhance the release of EVs from HIV-infected U1 and -uninfected U937 promonocytic cell lines. Additionally, METH has also been shown to increase the release in the U1 cell–derived EVs of HIV-Nef and intercellular adhesion molecule-1 (ICAM-1) protein cargoes. These EVs were also shown to cause clustering and syncytia formation in uninfected, naive monocyte-derived macrophages, thereby implicating a pathway by which METH potentiates HIV neuropathogenesis [43].

## Opiates

Opiates are chemical compounds, such as opium, morphine, and codeine, which are found in the poppy plant. Opiates reinforce their effects via the G protein–coupled receptor family — µ, δ, κ, and orphaninFQ/nociception receptors [44]. The abundant glial cells, the astrocytes, have been shown to release astrocyte-derived EVs (ADEVs) in response to morphine exposure. For example, morphine-stimulated ADEVs can be taken up and internalized by microglia, leading, in turn, to induction of long intergenic noncoding RNA (lincRNA)-Cox2 expression, involving the (TLR7)/Nuclear factor kappa-B signaling pathway. Activation of the TLR7/Nuclear factor kappa-B pathway resulted in down-regulation of the microglial phagocytic activity, thereby contributing to the development of neuropathology and associated cognitive decline [45]. Likewise, Morphine-ADEVs carrying miR-138 were also shown to activate the TLR7-NF-kB axis and promote the production and release of proinflammatory cytokines, Tumor Necrosis Factor-alpha/Interlukin 6, in microglia [46]. Additionally, findings from our lab have also shown brain region–specific increase in astrocytic amyloids in rhesus macaques chronically administered morphine. Mechanistically, exposure of human primary astrocytes to morphine resulted in upregulation of the hypoxia-inducible factor (HIF-1α), in turn, stimulating the sequential expression of the β-site cleaving enzyme (BACE1), Amyloid precursor protein, and toxic Aβ products. Furthermore, the morphine-mediated astrocytic amyloid cargo was shown to be propagated via a paracrine mechanism involving morphine-stimulated ADEVs, both *in vivo* and *in vitro*, ultimately leading to the progression of neuroinflammation [47].

## Alcohol

Alcohol use disorder is a chronic brain illness characterized by compulsive alcohol use despite its negative consequences [48]. Alcohol disrupts numerous neurotransmitter systems, thereby disturbing the fine balance between inhibitory and excitatory neurotransmitters [49]. Alcohol has been shown to modulate microglial cell cycle progression, exosome release, and exosome composition [50–52]. Additionally, alcohol-mediated induction of microglial EV release has been shown to involve altered lipid homeostasis and activation of mitochondria-associated endoplasmic reticulum membranes (MAMs) and sphingomyelinases (SMases) — an enzyme that catalyzes the hydrolysis of sphingomyelin into ceramide [53]. MAMs are specialized subdomains of the endoplasmic reticulum that resemble lipid rafts and play a crucial role in lipid metabolism [54]. Cholesterol and sphingomyelin are critical components for maintaining lipid raft integrity, with cholesterol levels regulated through crosstalk between the plasma membrane and the endoplasmic reticulum [55]. Cholesterol internalization and its transport to the endoplasmic reticulum stimulate MAM formation, activating SMases to facilitate cholesterol translocation and SMase-dependent EV release [56]. Ethanol enhances microglial cholesterol uptake and SMase activity, particularly neutral SMase, which facilitates ceramide production — a crucial step for the inward budding and shedding of intraluminal vesicles. This process promotes exosome formation through the ESCRT-independent pathway driven by neutral SMase activity [53]. Notably, SMase inhibitors, such as GW4869 and desipramine, block ethanol-induced EV secretion, highlighting their potential as therapeutic targets [53,57]. In astrocytes, ethanol through the TLR4 axis is shown to enhance EV release while also upregulating the expression of miRNAs (mir-146a, mir-182, and mir-200), as well as inflammatory proteins (TLR4, NFB-p65, IL-1R, caspase-1, NLRP3) in ADEVs, thereby contributing to neural cell death and neurodegeneration [58]. In a rat model of fetal alcohol spectrum disorders, ethanol exposure to postnatal rat pups resulted in increased expression of the microglial-specific protein CD13 (an aminopeptidase), as well as the matrix metalloproteinases-2 (MMP2: a marker of exosome activity) in exosomes collected from the mediobasal hypothalamus region, thus indicating ethanol-mediated potentiation of exosome release in microglia. In addition, these exosomes were also shown to be taken up by hypothalamic β-endorphin neurons, resulting in neuronal death. The underlying mechanism(s) in this process involve elevated expression of complement protein c1q in exosomes. It is likely that the accumulation of C1q in β-endorphin neurons could, in turn, activate the complement system and/or stimulate the production of reactive oxygen species, resulting in oxidative stress and cell death [57].

## Tobacco/Nicotine

Nicotine, an addictive neuroactive alkaloid present in tobacco plants, mediates its effect in the Central Nervous System through nicotinic acetylcholine receptors (nAChRs). Tobacco is primarily smoked but can also be chewed or inhaled [59]. Tobacco, much like opiates and cannabis, has been reported to alter the EV composition and release [60,61]. As an example, Koul et al. demonstrated sex-specific variation in brain-derived EV (BDEVs) biogenesis and EV cargo content in a rat model of nicotine self-administration. Furthermore, it was observed that BDEVs from male rats were smaller in size compared with those from females, with 31 proteins in males and almost 85 proteins in females that were differentially expressed. The differentially expressed proteins included proteins related to axon regeneration, glial cell proliferation, synaptic vesicles, and GABAergic synapses, thus implicating that nicotine could affect critical glial and neuronal functions [62]. Mechanistically, nicotine modulates axon regeneration through an nAChR-dependent pathway, characterized by upregulated nAChR expression and reduced receptor internalization. Nicotine exposure in mice has been shown to facilitate axonal regeneration [63,64]. Additionally, nicotine-mediated α7-nAChR activation is shown to suppress microglial activation, proliferation, and proinflammatory responses, reducing neuroinflammation while sparing astrocytes [65]. Nicotine also interacts with the synaptic vesicle protein SV2C to regulate dopamine release, α-synuclein proteostasis, and autophagy [66]. In the ventral tegmental area, nicotine-mediated enhancement of glutamatergic inputs to dopamine neurons involved the activation of α7-nAChR, in turn, modifying synaptic transmission [67,68]. These findings thus suggest that the enrichment of neuronal & glial protein cargoes in nicotine-BDEVs could modulate the programming of the recipient cells [62]. Furthermore, the exposure of cigarette smoke condensates (CSC) to HIV-infected U1 macrophages was shown to decrease the packaging of anti-inflammatory cytokines interleukin-10 (IL-10), while concomitantly increasing the packaging of proinflammatory cytokines, interleukin-1β (IL-1β), and interleukin-6 Tumor Necrosis Factor-alpha/Interlukin 6, in EVs. Furthermore, EVs originating from these cells could also transfer IL-1β to neurons and astrocytes, thereby enhancing their inflammatory status, which, in turn, could lead to astrocytosis and/or neuronal impairment/death [69,70]. A recent study reported that acute tobacco exposure in healthy nonsmokers stimulated the secretion of EVs from endothelial cells. How these endothelial cell–derived EVs contribute to neurodegeneration, however, remains unclear and warrants further investigation [71].

## Future perspectives

SUD is an escalating global health concern affecting millions of individuals worldwide and contributing to significant socio-economic and healthcare burdens. In this review, we have put together the current state of evidence suggesting the role of EVs in SUD pathogenesis. Overall, the existing literature suggests that substance use dysregulates cellular genes and protein expression. Additionally, it is evident from various studies that substance use can directly influence EV secretion; however, the mechanisms underlying altered EV cargo (miRNAs, mRNAs, lncRNAs, inflammatory cytokines, and synaptic proteins) release remain poorly understood. Future research should encompass an assessment of drug-specific EV-associated cargoes and their effects on neural communication, plasticity, and glial cells, for a comprehensive understanding of EV-mediated modulation of SUD pathogenesis.

## Data Availability

No data were used for the research described in the article.
